# Weight Cycling and Dieting Behavior in Fitness Club Members

**DOI:** 10.3389/fendo.2022.851887

**Published:** 2022-05-03

**Authors:** Lene A. H. Haakstad, Trine Stensrud, Gro Rugseth, Christina Gjestvang

**Affiliations:** Department of Sports Medicine, Norwegian School of Sports Sciences, Oslo, Norway

**Keywords:** body-mass index (BMI), dieting behavior, fitness clubs, novice exercisers, weight loss attempts

## Abstract

**Background:**

Along with the rising prevalence of high body-mass index (BMI), there is also increased emphasis on leanness and fitness. Both these trends suggest that many individuals are concerned about weight management and may try to lose weight. Using data from the research project “Fitness clubs - a venue for public health?”, we aimed to describe weight cycling and energy-restricted dieting in men and women at start-up of fitness club membership, and to investigate influencing factors [age, BMI, educational level, self-classified overweight/obesity, compliance with nutritional guidelines, unhealthy weight control strategies and self-perceived health (SPH)].

**Methods:**

In a cross-sectional online survey, 250 men and women from 25 fitness clubs in Oslo, reported anthropometrics, self-classified weight group, weight cycling, weight loss/gain, eating habits/dieting, and background/health information. Enrollment was limited to adult (≥18 years) novice exercisers (exercising <60 min/week at a moderate or vigorous intensity or brisk walking <150 min/week, the past six months) with less than four weeks of membership. Factors associated with weight cycling were examined using simple and multiple logistic regression, separated for men and women.

**Results:**

In both sexes (mean age: 36.4 ± 11.3, range 18-71 years), a high number reported substantial weight fluctuation (+/-5 kg) the past 12 months (men: 50% and women: 62%, mean difference 12%, 95% CI -0.3 to 23.8, p=0.056) and unhealthy weight control strategies (men: 24.8% and women: 47.2%, mean difference 22.4%, 95% CI 10.5 to 33.4, p<0.001). Weight cyclers had a higher mean BMI compared with non-cyclers (mean difference -1.5, 95% CI -2.6 to - 0.4, p= 0.003). Further, the difference in body weight was 6.7 kg (95% CI 2.2 to 10.8, p=0.004) and 10.8 kg (95% CI 5.8 to 15.8, <0.001) in men and women, respectively. Besides BMI status, self-classified overweight/obesity was the strongest predictor of reporting weight cycling (men: OR 5.54, 95% CI 2.03 to 15.12, p<0.01 and women: OR 7.17, 95% CI 2.48 to 20.68, p<0.001).

**Conclusion:**

In novice exercisers, a large proportion reported weight cycling and unhealthy weight control strategies, and both were more prevalent in women than in men. Self-classified overweight was found to be the most important factor influencing weight cycling.

## Introduction

High body-mass index (BMI) is a major risk factor for non-communicable diseases, such as cardiovascular disease, type 2 diabetes, high blood pressure, musculoskeletal complaints, some cancers and mental health challenges (e.g., depression) (1). High BMI is also associated with preventable premature death when looking at all-cause mortality (2–4). Also, in Scandinavia, the health and economic burden of the high prevalence of overweight (57.6%) and obesity (21.5%) has led to great concerns, and efforts to stop the progression are seen at several levels (national, educational, medical, community, parental, and individual) (2, 5, 6). Even if obesity has become more widespread, weight stigma has not subsided, and along with the rising prevalence of high BMI, there is also increased emphasis on leanness and fitness (7, 8). Ultimately, both these trends suggest that many individuals are preoccupied with weight concerns and may try to lose weight for both aesthetic and health reasons (5, 9).

Today, weight loss attempts have become so common that periodic dieting may be considered the new “normal” (10). In the United States 49% of all adults and 67% of adults with obesity have tried to lose body weight the last year (11). This is comparable with worldwide estimates of weight loss attempts, with the highest prevalence in Europe and Central Asia (about 61%) (12). Data from Norway showed that more than 50% of women were currently trying to alter their body weight and that weight loss attempts were very strongly associated with BMI (13, 14).

Current evidence supports moderate energy restriction in combination with exercise for weight loss in both men and women (15, 16). In adults trying to lose body weight, these were also the two most frequently reported approaches (exercising 62.9% and eating less food 62.9%) (11). Yet, concerns have been raised about the focus on body weight in the society, and public health efforts to slow down the obesity pandemic have not been successful in the past 40 years (5, 17). In fact, repeated attempts to lose weight are shown to be associated with weight cycling, known as yo-yo dieting, coined by the public health scholar Kelly D. Brownell (17). Repeated loss and regain of body weight may also contribute to excessive weight gain, body fat and added health risks (10). In the present study, a substantial bodyweight cycling was defined as +/- 5 kg the past year, which agrees with the National Institute of Health (18).

In fitness clubs, exercise in combination with energy-restricted diets (eating fewer calories than normal) are often promoted as a strategy for weight loss management. Also, one of the main reasons that lead adults to join such a club involve weight loss (19, 20). According to IHRSA (2020), fitness clubs have about 185 million members worldwide, representing a 54% increase over the last decade (21). In Norway, around 30% are members of a gym, which is at the very top in Europe (22). Although weight loss attempts and yo-yo dieting may be common phenomenon’s in novice exercisers, no research has been carried out in the social context of a fitness club.

Low self-perceived health (SPH) has been linked to overweight/obesity and irregular exercise (23–25). Still, the association between weight cycling and SPH remains to be elucidated. SPH is a single item measure considering both somatic and psychological health (26, 27). SPH also reflects a normative component, and the belief of how one should act to be healthy (27). Using data from the research project “Fitness clubs - a venue for public health?”, we aimed to describe weight cycling and energy-restricted dieting in men and women starting a fitness club membership, and to investigate influencing factors (age, BMI, educational level, self-classified overweight/obesity, compliance with nutritional guidelines, unhealthy weight control strategies and SPH), which has earlier not been examined in this population. As weight loss is reported to be one of the main reasons why adults join fitness clubs, we expected that we would find a high proportion of weight cyclers in new club members, especially among female participants with a high BMI.

## Materials and Methods

### Design and Participants

The cross-sectional data collected in this study was part of a longitudinal prospective study conducted in Oslo (Norway) from October 2015 to October 2018, following a group of novice exercisers at 25 fitness clubs in Oslo, Norway (19, 28). The primary aim of the original project was to gain an increased understanding of those individuals who are able to stay active and continue with regular exercise in a group of novice exercisers in their first year of fitness club membership. Participants were recruited by an e-mail invitation from their local fitness club (SATS chain). In this e-mail, the aims and implications of the study were explained. Among those who expressed interest in participating in the study, the eligibility criteria were checked by a follow-up email or phone call from our research fellow. At first contact, they were also informed that they could contact the primary researcher if they had any questions about participation, or if they had any issues regarding answering the questionnaire, such as understanding the survey structure. The participants had to be healthy novice exercisers (≥18 years), with <four weeks membership. Healthy was defined as no disease considered to hinder physical activity (such as heart disease, severe hypertension, or asthma), and novice exercisers were defined as <60 min/week of exercise at moderate or vigorous intensity, or brisk walking <150 min/week, in the last six months (28). Hence, all were considered non-exercisers at start-up, including baseline measures investigating weight cycling and dieting behaviour. In total, 676 fitness club members wanted to participate in the study. We excluded those who already exercised regularly (n = 270) or had cardiovascular disease, severe hypertension, or asthma (n = 8). Besides, 148 individuals did not respond after the first e-mail. Hence, 250 fitness club members (equal number of men and women) were included and completed the baseline electronic questionnaire at start-up at the fitness club.

### Ethical Approval

The Regional Committee for Medical and Health Research Ethics, Southern Norway, Oslo, revised the project and complete data collection (REK 2015/1443 A) and concluded that the study did not require full review according to the Act on medical and health research (the Health Research Act 2008). All participants signed an informed consent form, following the Helsinki declaration. The study was approved by the Norwegian Social Science Data Service (NSD 44135) and was financed by a PhD position (CG) at the Norwegian School of Sports Sciences (NSSS). Data was non-identifiable, and confidentiality was maintained in accordance to the law. Participation in the project did not involve any harmful or invasive investigations. It was emphasized that participation was voluntary and that everyone who chose to participate could withdraw partially or fully from the project at any time without further explanation. No economic compensation was given to the participants. The IT department at NSSS provides storage services, and Norwegian regulations require that all raw research data should be kept for at least five years after study completion.

### Measures

The standardized electronic questionnaire (SurveyXact, www.survey-xact.no) contained 52 questions, took about 25 minutes to complete and were outlined as either placing a check mark (√), Likert scales assessing level of agreement to a statement or fill in the blank. Since it may be unethical with mandatory questionnaire responses, the participants could tick *“Does not apply”* or *“I do not want to answer”* as a response option on all questions. The questionnaire was piloted for comprehensibility of questions and answer options among four members in the research group and four volunteers. Motives for fitness club participation and exercise, have previously been reported in a separate publication (19), which was based on the validated questionnaire ‘Reasons for Exercise Inventory’ (Exercise Motivations Inventory—2 (EMI-2). Below are the questions used to answer the present research questions. Some have been used in a Danish study among fitness club (29), and we have previously showed that new gym members reported their body weight and height with reasonable accuracy, and that subsequent BMI may be a valid measure among both sexes (30):

1. Body weight and weight cycling:
  - *“What is your current weight in kilograms (kg)?”: ______*
  - *“What is your height in centimeters (cm)?”:* ______
  - *“Have you had body weight cycling +/- 5 kg the past year”.* Response options: *“Yes”* or *“No”*
  - *If you had any weight change the past year, what was the size of your weight change?* Response options: *“Weight loss (in kilograms, kg)”: ______* or “*Weight gain* (*kilograms, kg)”: ______*
  - *On a scale from 1-5*, *where 1 represents “Very dissatisfied” and 5 represents “Very satisfied, how satisfied are you with your body weight?* High satisfaction was defined as a score ≥ 4.
  - Self-classified weight group: *“I think I am…”* The response options were grouped according to World Health Organization (WHO) BMI classification: *“underweight (<18.5 kg/m^2^)”, “normal weight (18.5 to 24.9 kg/m^2^)”, “overweight (25 to 29.9 kg/m^2^)” and “obese (≥ 30 kg/m^2^)”.*
2. Eating habits and energy-restricted dieting:
  - The Norwegian directorate of health recommend a balanced and varied diet, comprised of whole grain products, vegetables, fruits and berries, lean dairy products, fish, legumes and nuts, while also limiting the amount of processed meats, red meat and foods high in saturated fat, sugar and salt. *With this in mind, how would you characterize your diet last four weeks?* The participants rated their diet on a scale from 0-10, where 0 represented *“Very poor”* and 10 represented *“Very good”.* Good nutritional habits and compliance with nutritional guidelines was defined as a score ≥ 7 on an 11-point scale.
  - *“Are you a vegetarian”* Response options: *“Yes”* or *“No”*
  - *“Have you tried to lower daily caloric intake for example by missing meals or fasting?”* Response options*: “Never”, “Rarely”, “Sometimes”, “Often”* or *“Very often.* Participants answering “Sometimes”, “Often” or “Very often” was categorized as using unhealthy weight control strategies.
3. Self-perceived health (SPH): *“In general, how would you rate your health today?”.* Response options: *“Excellent”*, *“Good”, “Moderate”, “Fair”* and *“Poor”* (26). According to these five selections, participants were also divided into two categories: high SPH (“Excellent” and “Good”) and low SPH (“Moderate, “Fair” and “Poor”), an approach adopted by other researchers (31).
4. Demographic information: *age, sex, education, ethnicity, cohabitation, employment, household income, children, smoking* and *sick leave.*

### Statistical Analysis

All statistics were conducted with IBM SPSS Statistics 28.0. Descriptive data were screened for normality and outliers, including a comparison of the overall curve of the bars of the histograms, and the usage of parametric statistics. For descriptive purposes, the results are shown as numbers with percentages or means with standard deviations (SD), as well as group differences and Odds Ratio (OR) with 95% Confidence Intervals (CI) and p-values. For ORs less than 1, we chose to invert these (1 divided by the actual value) to aid interpretation (32).

In agreement with the National Institute of Health (18), a substantial bodyweight cycling was defined as +/- 5 kg the past year. The associations between weight cycling (dichotomous dependent variable, coded 0/1) and the predictive power of seven independent selected variables (age, BMI, educational level, self-classified overweight/obesity, compliance with nutritional guidelines, unhealthy weight control strategies and SPH) were analyzed using simple and multiple logistic regression, separately for men and women. Age and BMI were treated as a continuous variable, whereas all other variables were categorized, using one category as the reference group. Level of statistical significance was set at p < 0.05.

## Results

### Participants Characteristics

The mean age was 36.4 ( ± 11.3, range 18-71) years, and the majority were of Norwegian descent (78.4%). Individuals with university education (75.2% of the participants versus 35.6% of the Norwegian population) were overrepresented compared with high-school education (22.0% of the participants versus 37.1% of the Norwegian population) (33). In 36.8%, mean household income was classified as “high” (>$100 000 per year), and 55.4% were employed full-time. Cohabitation/marriage was reported by 61.2%, and 32.0% had children. We found a higher mean age in men compared with women (38.4 years versus 34.3 years, mean difference 4.1 years, 95% CI 6.9 to 1.3, p=0.002) and a tendency towards higher mean household income (42.4% versus 31.2%, mean difference 11.2%, 95% CI -0.7% to 22.7%, p=0.066). Otherwise no other differences by sex were observed in background characteristics of the sample.

### BMI, Self-Selected Weight Group, and Weight Cycling

Self-reported body weight cycling during the previous 12 months, BMI measures and self-selected weight group by sex are shown in **Table 1**. The prevalence of overweight and obesity (BMI≥25) was higher than the corresponding self-selected weight group for men (61.6% versus 47.2%, mean difference 14.4%, 95% CI 2.1 to 26.1, p=0.034) and almost consistent for women (40.0 versus 46.4%, mean difference 6.4%, 95% CI -5.8 to 18.3, p=0.308). A high number in both sexes reported weight cycling (+/-5 kg) the past 12 months (men: 50% and women: 62%, mean difference 12%, 95% CI -0.3 to 23.8, p=0.056). About 40% had gained ≥10 kg, and 27% had lost ≥10 kg the last year, with no sex differences. When comparing individuals with university and high school education, we found no difference in reports of weight cycling (44.1% versus 50.0%, p=0.420).

**Table 1:** Participants reporting body weight cycling during the previous 12 months, BMI measures and self-selected weight group by sex (n=250).

Variables	Men (n=125)	Women (n=125)	Group difference (95% CI)	p-value
**n (%)**				
Body weight cycling +/- 5 kg the past year	62 (49.6)	77 (61.6)	12.0 (-0.3 to 23.8)	0.056
Excessive weight gain				
- ≥ 5 kg	38 (61.3)	50 (64.9)	3.6 (-8.3 to 15.3)	0.662
- ≥10 kg	15 (24.2)	19 (24.7)	0.5 (-10.1 to 11.1)	0.946
- ≥ 15 kg	6 (9.7)	9 (11.7)	2 (-5.9 to 9.9)	0.707
- ≥ 20 kg	4 (6.5)	4 (5.2)	1.3 (-4.9 to 7.7)	0.745
Excessive weight loss				
- ≥ 5 kg	24 (38.7)	27 (35.1)	3.6 (-8.3 to 15.3)	0.663
- ≥ 10 kg	10 (16.1)	11 (14.3)	1.8 (-7.2 to 10.8)	0.769
- ≥ 15 kg	5 (8.1)	5 (6.5)	1.6 (-5.2 to 8.5)	0.718
- ≥ 20 kg	4 (6.5)	3 (3.9)	2.6 (-3.3 to 8.8)	0.486
BMI groups (kg/m^2^)				
- Underweight (<18.5)	**-**	1 (0.8)	–	–
- Normal weight (18.5 – 24.9)	48 (38.4)	74 (59.2)	24.4 (12.1 to 35.7)	0.002
- Overweight (25.0-29.9)	57 (45.6)	34 (27.2)	18.4 (6.5 to 29.6)	0.003
- Obese (≥30)	20 (16.0)	16 (12.8)	3.2 (-5.6 to 12.0)	0.471
Self-classified weight group*				
- Underweight	2 (1.6)	2 (1.6)	–	1.0
- Normal weight	64 (51.2)	64 (51.2)	–	1.0
- Overweight	53 (42.4)	48 (38.4)	4.0 (-8.1 to 15.9)	0.520
- Obese	6 (4.8)	10 (8.0)	3.2 (-3.2 to 9.8)	0.302
**Mean (SD)**				
Body weight (kg)	86.4 (12.7)	70.9 (14.9)	-13 (-17.1 to -10.3)	<0.001
Body height (m)	1.81 (0.8)	1.68 (0.6)	-0.13 (-0.3 to 0.04)	0.147
BMI (kg/m^2^)	26.2 (3.7)	25.0 (5.0)	-1.2 (-2.3 to -0.1)	0.026
Weight gain past 12 months (kg)	4.0 (6.5)	5.2 (6.6)	-1.2 (-2.8 to 0.4)	0.250
Weight loss past 12 months (kg)	2.8 (5.9)	2.7 (5.0)	-0.1 (-1.5 to 1.3)	0.899

* “I think I am…” (underweight, normal weight, overweight, obese).

Group differences are presented as percentages or variables units.

With reference to any weight change the past year (small or large), in women, mean weight gain was higher (5.2 kg) than mean weight loss (2.7 kg) (mean difference + 2.5 kg, 95% CI 1.04 to 3.96, p<0.001). In men, the difference in weight gain and weight loss was +1.2 kg (CI 0.34 to 2.75, p=0.128).

The distribution of “no weight change”, “weight gain” and “weight loss” according to self-selected weight group are shown in **Table 2**. No weight changes in the past 12 months were reported by a higher number of normal weight than overweight or obese participants (75.0% versus 31.5%, mean difference 43.5%, 95% CI 25.6 to 57.7, p<0.001). Further, weight gain was more prevalent than weight loss in the two highest weight groups. In both sexes, cyclers weighed more (82.9 kg versus 75.1 kg, mean difference 7.8 kg, 95% CI 11.6 to 4.0, p<0.001) and had a higher mean BMI compared with non-cyclers (27.0 ± 4.9 versus 25.4 ± 3.6, mean difference 2.6, 95% CI 1.6 to 3.6, p= 0.003). Among women, the difference in body weight was 10.8 kg (CI 5.8 to 15.8, <0.001), whereas for men, the difference was 6.7 kg (CI 2.2 to 10.8, p=0.004) in disfavor of cyclers. Treating BMI as a continuous variable and expressing the associations between BMI and weight cycling (dichotomous dependent variable) in terms of odds ratios (ORs), gave an OR of 1.17 (CI 1.08 to 1.25, p<0.001). Hence, for each unit change in BMI, the odds of reporting substantial weight cycling increased by 1.17.

**Table 2:** Descriptive data showing distribution of “no weight change”, “weight gain” and “weight loss” in new fitness club members the past 12 months according to self-selected weight group (n=250)*.

Self-selected weight group**	No weight change	Weight gain	Weight loss
Underweight (n=4)			
- n (%)	0	2 (50.0)	2 (50.0)
- Mean (SD)	–	3.0 (4.2) kg	5.5 (0.7) kg
Normal weight (n=138)			
- n (%)	96 (75.0)	23 (16.7)	19 (13.7)
- Mean (SD)	–	1.9 (4.0) kg	2.1 (4.7) kg
Overweight (n=101)			
- n (%)	32 (31.7)	59 (58.4)	32 (31.7)
- Mean (SD)	–	6.4 (6.8) kg	3.2 (6.1) kg
Obese (n=16)			
- n (%)	5 (31.3)	10 (62.5)	4 (25.0)
- Mean (SD)	–	12.5 (9.5) kg	3.8 (6.1) kg

*Several participants reported both weight gain and weight loss the past 12 months, hence the numbers don’t add up to 100% in each weight group category.

**“I think I am…” (underweight, normal weight, overweight, obese).

Among those who self-selected themselves as overweight or obese, only two (one man and one woman) were satisfied with their body weight. Regardless of weight status, 75.6% were dissatisfied with bodyweight with no sex differences. The majority of those reporting weight cycling the past year, both women and men, were dissatisfied with body weight (50.8% versus 29.5%, mean difference 21.3%, 95% CI 9.1 to 32.5, p=0.004), and women rated their SPH somewhat lower than non-cyclers (2.14 versus 2.38, mean difference -0.24, 95% CI -0.51 to 0.03, p=0.079).

### Eating Habits and Energy-Restricted Dieting

In both sexes, less than one fourth complied with nutritional guidelines. On a scale from 0-10, women rated their diet and nutritional habits slightly better than men (6.2 ± 2.0 versus 5.5 ± 2.6, p=0.023). Also, normal weight women rated their diet better than those who perceived themselves as overweight/obese (6.6 ± 1.9 versus 5.6 ± 2.0, mean difference -1.0, 95% CI -1.7 to -0.3, p=0.004). This difference was not observed in men. Few (2.0%, no sex differences) reported vegetarianism, which is about the same as the general adult population in Norway (3.0%) (34).

Men and women reporting energy-restricted dieting (such as missing meals and fasting) are shown in **Table 3**. In both sexes, a high percentage reported unhealthy weight control strategies to lower daily caloric intake, and women were more likely to report such practice than men. Mean BMI was higher in those using unhealthy weight control strategies than those not reporting this (men: 27.4 ± 3.9 versus 25.8 ± 3.5, mean difference -1.6, 95% CI -3.1 to -0.1, p= 0.053 and women: 26.0 ± 5.7 versus 24.2 ± 4.1, mean difference -1.8, 95% CI -3.4 to -0.6, p= 0.046), and the prevalence for unhealthy weight control strategies were 33.3% in normal weight, 32.2% in overweight and 55.6% among obese participants (mean difference between obese and normal weight/overweight 22.8%, 95% CI 5.5 to 38.8, p=0.058).

**Table 3:** Energy-restricted dieting and prevalence using unhealthy weight control strategies (missing meals and fasting) to lower daily caloric intake by sex (n=250).

n (%)	Men (n=125)	Women (n=125)	Group difference (95% CI)	p-value
Missing meals and fasting to lower daily caloric intake				
- Very often	2 (1.6)	2 (1.6)		
- Often	3 (2.4)	9 (7.2)		
- Sometimes	26 (20.8)	48 (38.4)		
- Rarely	21 (16.8)	29 (23.2)		
- Never	73 (58.4)	37 (29.6)		
Unhealthy weight control strategies*				
- Yes	31 (24.8)	59 (47.2)	22.4 (10.5 to 33.4)	<0.001
- No	94 (75.2)	66 (52.8)		

*Participants answering “Sometimes”, “Often” or “Very often” was categorized as using unhealthy weight control strategies.

Based on self-classified weight group, 13.6% of men viewing themselves as underweight/normal weight and 37.3% as overweight/obese (mean difference 23.7%, 95% CI 8.5 to 37.9, p=0.002) reported unhealthy weight control strategies, whereas the corresponding number for women were 36.4% and 60.3% (mean difference 23.9%, 95% CI 6.3 to 39.6, p=0.008).

In both sexes, weight cycling showed no association with the use of unhealthy weight control strategies (men, p=0.437 and women, p=0.665), age (men, p=0.643 and women, p=0.443), educational level (men, p=0.615 and women, p=0.815), diet or SPH (men, p=0.648 and women, p=0.227), and therefore not included in the final logistic regression model. The strongest predictor of reporting weight cycling was self-classified overweight/obesity, showing an OR of 5.54 (95% CI 2.03 to 15.12, p<0.01) in men and 7.17 (95% CI 2.48 to 20.68, p<0.01) in women.

## Discussion

Dieting behavior and weight cycling may contribute to excessive weight gain and added health risks. Hence, it is important to study weight cycling, the use of unhealthy weight control strategies, and how well individuals perceive overweight and obesity. To our knowledge, this is the first study to report weight cycling and energy-restricted dieting by weight status and sex in a fitness club setting. Our results showed that half of the men and more than 60% of the women reported substantial weight cycling the past 12 months, whereas unhealthy weight control practices were reported by one-fourth of the men and half of the women. Hence, both practices are quite common in both sexes, with surprisingly 40% gaining ≥10 kg, and 27% losing ≥10 kg the last year. Female cyclers weighed nearly 11 kg more than non-cyclers, whereas for men the difference was close to 7 kg. In both sexes, self-classified overweight/obesity was the strongest predictor of reporting weight cycling. Since a high BMI has become more widespread, overweight and obesity will likely continue to grow, given the fact that so many individuals are encouraged to attempt to lose weight.

The present study findings are somewhat higher compared with previous reports, showing prevalence numbers in weight cycling from 20% to 35% in men and 20% to 55% in women (35, 36). A challenge in the interpretation of results is the variability in recruited participants and that there is no clear definition of weight cycling. Also, there is inconsistency in the amount of mean weight lost and regained, and the number of weight loss attempts during different periods (35). For instance, in a study of women with more than 46 000 participants, intentional weight cycling varied from one to three times the last four years, and the amount of weight lost ranged from about 2 kg to 4.5 kg (37). We asked our participants about weight cycling within the past year and categorized substantial body weight cycling as +/- 5 kg. With respect to mean weight loss, this was below 3 kg in both sexes, whereas mean weight gain was 4 kg in men and more than 5 kg in women. An interesting result is the surprisingly high number reporting that they had gained (40%) or lost (27%) more than 10 kg the last 12 months, indicating that both excessive weight gain and weight loss are quite frequently reported by new fitness club members. To the best of our knowledge, no previous studies have investigated this in a fitness club setting.

In US and Europe, about 50% to 60% of all adults have tried to loose weight last year (11, 12), with the highest prevalence in women. Data from Norway showed that more than 50% of women were currently trying to alter their body weight (13, 14). However, prospective studies suggest that few are successful in maintaining weight loss, exposing individuals to weight cycling with repeated weight loss attempts (35, 38). According to the Norwegian Institute of Public Health, overweight and obesity have steadily increased since 1975, and average BMI (kg/m^2^) is now about 26 (overweight category) compared with former 23 (normal weight category) (39). We found corresponding numbers in the present study sample, with a BMI of 26.3 in men and 25.0 in women. Worldwide, weight management is an important public health challenge and women seem to be exposed to weight cycling to a greater degree than men (35, 40). Sex differences were also evident in the present study, and prevalence of weight cycling the past 12 months reached 60% of the women compared to 50% of the men. There is some evidence that women (from adolescents years) may be more susceptible to societal expectations of leanness and ideal body size than men (40, 41), and in our study twice as many women compared with men reported unhealthy weight loss strategies and energy-restricted dieting such as missing meals and fasting. On the other side, they also rated their diet and healthy eating habits slightly better than men, which is in accordance with other reports (42). Regrettably, we did not collect data about binge eating behaviour, which could have brought some insight into the mechanisms of weight cycling (43). A recent review has shown that women report higher levels of clinical and subclinical types of eating disorders, including binge eating and loss-of-control eating (44). We did, however, ask the participants about SPH, and lower SPH has been linked to overweight/obesity and binge-eating disorder (24, 45). Because women, as compared with men, often consider a broader set of aspects when rating their health (such as mild health complaints and psychological factors), sex differences in SPH are often reported (46, 47). In the present study, no difference in SPH was found between men and women, but female weight cyclers reported slightly lower SPH than non-cyclers. The complexity of weight-cycling highlights the need for future qualitative studies and high-quality randomized controlled trials, investigating this in more depth, allowing for a further understanding of how we should target weight management.

As for the prevalence of weight cycling according to self-classified weight status, the study results are beyond what we had anticipated. In men and women, those with self-classified overweight/obesity were 5.5 and 7.2 times more likely to report weight cycling the past year compared with normal weight individuals. Yet, perceived overweight/obesity was not associated with healthy eating or the use of healthy or unhealthy weight strategies. In the literature, rather than being associated with improved weight management, perceived overweight (in both sexes, and among adults and children) is consistently shown to predict weight gain over time (17, 25). Individuals who perceive their weight status as overweight are more likely to report weight loss attempts, driving the “weight loss futile cycle”, associated with many adverse health outcomes (17). An important question is therefore whether weight loss should be the primary focus of overweight and obesity treatment. The current public health approach has been largely ineffective and has not been able to reverse the obesity pandemic (5, 17). In fitness clubs, exercise is often promoted as a strategy for weight loss management. Also, one of the main reasons that lead adults to join such a club involves weight loss (19, 20). We have previously reported motivational factors for fitness club participation in a separate publication (19), and found that main motives for fitness club membership and exercise were related to physiological factors, with increase in physical fitness (92.8%) most frequently cited, followed by health-related motives. Appearance and weight management reasons were reported by about 50% of the participants.

Maintaining weight loss has been repeatedly established as extremely difficult, explained by both the physiological responses from the body to calorie restrictions, combined with the physical and mental effort of a rapid increase in heavy exercise (48). We would, therefore, put emphasis on a weight neutral instead of weight loss approach when targeting health and fitness in gym members. This strategy could avoid the pitfalls associated with repeated weight loss failure (49, 50). Research has shown that physical activity and improvements in cardiorespiratory fitness may counterbalance the risks associated with a high BMI and adiposity, regardless of weight loss (51–53). In addition, increasing physical activity level is consistently shown to give a greater reduction in cardiovascular disease and all-cause mortality than intentional weight loss. It can, however, be questioned if the capacity to exercise is reduced in individuals with obesity. In our participant group, equally distributed in men and women, about 14% were categorized with a BMI≥30, and three women had severe obesity (BMI≥40.0). Also, we recognize that it is challenging to promote exercise training without a weight loss goal in a fitness club (e.g. personal trainer), considering that more than half of the adult population want to lose weight (12, 13, 54). This group might, however, be more willing to adopt health-enhancing behavior and regular exercise without a weight loss focus, if personal trainers/fitness instructors and health care providers highlighted this perspective and promoted the numerous health benefits associated with exercise without weight loss.

### Strength and Limitations

Several studies have reported prevalence of weight cycling in different adult populations, yet to our knowledge, this is the first study to describe weight cycling and energy-restricted dieting in novice exercisers starting a gym membership. Other aspects were a complete dataset, with no missing values, and that our participants included an age-diverse group of both sexes. The use of an electronic questionnaire to gather responses quickly and eliminate the costs associated with printing and distributing paper-based questionnaires may also be considered a strength. We recruited from 25 fitness clubs, and the sample (novice exercisers) is a study population of which there is limited knowledge. Although body weights and heights were derived from self-reports, a previous study has shown that BMI (kg/m2) based on self-report appears to be a valid measure in fitness club members (55). Also, our BMI data appears to be comparable to average BMI values in men and women in Norway (39). Finally, the inclusion of data concerning demographics and personal health variables made us able to investigate weight cycling and self-classified weight group, adjusting for recognized confounders (age, BMI, educational level, compliance with nutritional guidelines, unhealthy weight control strategies and SPH).

Limitations are the cross-sectional nature of the questionnaire, limiting directionality and causality between weight cycling and self-classified overweight/obesity. Also, this study depends on retrospective reporting of weight loss and weight gain, including the exact value of kg gained and lost. Even if this is common in the literature, it is questionable how correctly individuals recall such data, especially when considered over the last 12 months. In addition, we did not ask about the number of cycles of weight loss-regain that had occurred in the last year. There is reason to believe that weight suppression and history of dieting predict weight cycling for different reasons. As such, if we should have done the study protocol once more, we would have included the Revised Restraint Scale (56), a 10-item measure of dietary concerns and weight fluctuations.

Our population had little ethnic diversity, and data were obtained from one fitness club chain, with middle to high monthly costs, limiting the generalizability of findings. Enrollment of other clubs (such as low-cost gyms and CrossFit centers) might have given other results. Precautions in extrapolating the findings to other populations also need to be taken, because our participants included persons who were interested in health-enhancing behavior, exercise, and fitness. Also, precautions need to be taken in making comparisons between the sexes, because men and women may choose to join a fitness club for different reasons. Lastly, our quantitative cross-sectional data-collection with numeric results may be too narrow to explain the complex aspect of weight cycling. Hence, there is a need for future longitudinal research design, including both quantitative and qualitative data to assess temporality and exploring this behavior more in-depth.

### Conclusions

In novice exercisers, half of the men and 60% of the women reported weight cycling. Unhealthy weight control practices, such as skipping meals and fasting were reported by one-fourth of the men and half of the women. Hence, both practices are quite common, and this is a cause for concern. In both sexes, self-classified overweight/obesity was the strongest predictor of reporting weight cycling. Since a high BMI has become more widespread, overweight and obesity will likely continue to grow, given the fact that so many individuals attempt to lose weight. Hence, we need to shift from weight loss to a weight-neutral approach when targeting health and fitness in gym members. This strategy might help to avoid the pitfalls associated with repeated weight loss failure.

## Data Availability Statement

The raw data supporting the conclusions of this article will be made available by the authors, without undue reservation.

## Ethics Statement

The studies involving human participants were reviewed and approved by the Regional Committee for Medical and Health Research Ethics, Southern Norway, Oslo, revised the project and complete data collection (REK 2015/1443 A) and concluded that the study did not require full review according to the Act on medical and health research (the Health Research Act 2008). The study was approved by the Norwegian Social Science Data Service (NSD 44135) and was financed by a PhD position (CG) at the Norwegian School of Sports Sciences (NSSS). No economic compensation was given to the participants. The patients/participants provided their written informed consent to participate in this study.

## Author Contributions

LH conceived the idea for the research project and wrote the protocol together with CG. CG and LH was responsible for participant follow-up and data collection. LH supervised the project and outlined the manuscript. GR, TS, and CG contributed to interpretation of data, and revised it critically for important intellectual content, including English editing. All authors read and corrected draft versions of the manuscript and approved the final version.

## Funding

This work was supported by the Norwegian School of Sport Sciences, Department of Sport Medicine, Norway, and did not receive any specific grant from funding agencies in the public, commercial, or not-for-profit sectors.

## Conflict of Interest

The authors declare that the research was conducted in the absence of any commercial or financial relationships that could be construed as a potential conflict of interest.

## Publisher’s Note

All claims expressed in this article are solely those of the authors and do not necessarily represent those of their affiliated organizations, or those of the publisher, the editors and the reviewers. Any product that may be evaluated in this article, or claim that may be made by its manufacturer, is not guaranteed or endorsed by the publisher.
